# Use of 13-Valent Pneumococcal Conjugate Vaccine and 23-Valent Pneumococcal Polysaccharide Vaccine Among Adults Aged ≥65 Years: Recommendations of the Advisory Committee on Immunization Practices (ACIP)

**Published:** 2014-09-19

**Authors:** Sara Tomczyk, Nancy M. Bennett, Charles Stoecker, Ryan Gierke, Matthew R. Moore, Cynthia G. Whitney, Stephen Hadler, Tamara Pilishvili

**Affiliations:** 1 Epidemic Intelligence Service, CDC; 2 National Center for Immunization and Respiratory Diseases, CDC; 3 Pneumococcal Vaccines Working Group, Advisory Committee on Immunization Practices; 4 School of Medicine and Dentistry, University of Rochester Medical Center, Rochester, New York; 5 Department of Global Health Systems and Development, Tulane University of Public Health and Tropical Medicine, New Orleans, Louisiana

On August 13, 2014, the Advisory Committee on Immunization Practices (ACIP) recommended routine use of 13-valent pneumococcal conjugate vaccine (PCV13 [Prevnar 13, Wyeth Pharmaceuticals, Inc., a subsidiary of Pfizer Inc.]) among adults aged ≥65 years. PCV13 should be administered in series with the 23-valent pneumococcal polysaccharide vaccine (PPSV23 [Pneumovax23, Merck & Co., Inc.]), the vaccine currently recommended for adults aged ≥65 years. PCV13 was approved by the Food and Drug Administration (FDA) in late 2011 for use among adults aged ≥50 years. In June 2014, the results of a randomized placebo-controlled trial evaluating efficacy of PCV13 for preventing community-acquired pneumonia among approximately 85,000 adults aged ≥65 years with no prior pneumococcal vaccination history (CAPiTA trial) became available and were presented to ACIP (1). The evidence supporting PCV13 vaccination of adults was evaluated using the Grading of Recommendations, Assessment, Development, and Evaluation (GRADE) framework and determined to be type 2 (moderate level of evidence); the recommendation was categorized as a Category A recommendation (2). This report outlines the new recommendations for PCV13 use, provides guidance for use of PCV13 and PPSV23 among adults aged ≥65 years, and summarizes the evidence considered by ACIP to make this recommendation.

*Recommendations for routine use of vaccines in children, adolescents, and adults are developed by the Advisory Committee on Immunization Practices (ACIP). ACIP is chartered as a federal advisory committee to provide expert external advice and guidance to the Director of the Centers for Disease Control and Prevention (CDC) on use of vaccines and related agents for the control of vaccine-preventable diseases in the civilian population of the United States. Recommendations for routine use of vaccines in children and adolescents are harmonized to the greatest extent possible with recommendations made by the American Academy of Pediatrics (AAP), the American Academy of Family Physicians (AAFP), and the American College of Obstetrics and Gynecology (ACOG). Recommendations for routine use of vaccines in adults are harmonized with recommendations of AAFP, ACOG, and the American College of Physicians (ACP). ACIP recommendations adopted by the CDC Director become agency guidelines on the date published in the* Morbidity and Mortality Weekly Report (MMWR)*. Additional information regarding ACIP is available at http://www.cdc.gov/vaccines/acip.*

## Epidemiology of Pneumococcal Disease Among Adults Aged ≥65 Years

*Streptococcus pneumoniae* (pneumococcus) remains a leading infectious cause of serious illness, including bacteremia, meningitis, and pneumonia, among older adults in the United States. Use of a 7-valent pneumococcal conjugate vaccine (PCV7) since 2000 and PCV13 since 2010 among children in the United States has reduced pneumococcal infections directly and indirectly among children, and indirectly among adults. By 2013, the incidence of invasive pneumococcal disease (IPD) caused by serotypes unique to PCV13 among adults aged ≥65 years had declined by approximately 50% compared with 2010, when PCV13 replaced PCV7 in the pediatric immunization schedule (3). However, in 2013 an estimated 13,500 cases of IPD occurred among adults aged ≥65 years (3). Approximately, 20%–25% of IPD cases and 10% of community-acquired pneumonia cases in adults aged ≥65 years are caused by PCV13 serotypes and are potentially preventable with the use of PCV13 in this population (3,4).

## PCV13 Vaccine in Adults

On December 30, 2011, PCV13 was approved for use among adults aged ≥50 years to prevent pneumonia and invasive disease caused by *S. pneumoniae* serotypes contained in the vaccine. The new use for Prevnar 13 was approved under FDA’s accelerated approval pathway, which allows for earlier approval of products that provide meaningful therapeutic benefit over existing treatments for serious and life-threatening illnesses (5). FDA defined “meaningful therapeutic benefit over existing treatments” as protection of adults aged ≥50 years from nonbacteremic pneumococcal pneumonia or nonbacteremic pneumococcal pneumonia combined with protection from IPD (7). On June 20, 2012, ACIP recommended routine use of PCV13 for adults aged ≥19 years with immunocompromising conditions, functional or anatomic asplenia, cerebrospinal fluid leak, or cochlear implants (6). The ACIP decision to recommend PCV13 use among adults aged ≥65 years was deferred until data became available on 1) the impact of PCV13 use in children on disease in adults (i.e., indirect effects) and 2) the efficacy of PCV13 against noninvasive pneumococcal pneumonia among adults. In accordance with accelerated approval requirements, a randomized placebo-controlled trial (CAPiTA trial) was conducted in the Netherlands among approximately 85,000 adults aged ≥65 years during 2008–2013 to verify and describe further the clinical benefit of PCV13 in the prevention of pneumococcal pneumonia (1). The results of the CAPiTA trial demonstrated 45.6% (95% confidence interval [CI] = 21.8%–62.5%) efficacy of PCV13 against vaccine-type pneumococcal pneumonia, 45.0% (CI = 14.2%–65.3%) efficacy against vaccine-type nonbacteremic pneumococcal pneumonia, and 75.0% (CI = 41.4%–90.8%) efficacy against vaccine-type IPD among adults aged ≥65 years (1).

Two randomized, multicenter, immunogenicity studies conducted in the United States and Europe among older adults showed that PCV13 induced an immune response as good as or better than that induced by PPSV23 (7,8). Functional antibody responses were measured 1 month after vaccination using an opsonophagocytic activity (OPA) assay. In adults aged 60–64 years with no prior pneumococcal vaccination, PCV13 elicited OPA geometric mean antibody titers (GMTs) to the 12 serotypes common to both vaccines that were comparable with, or higher than, responses elicited by PPSV23 (7). In adults aged ≥70 years who previously had been immunized with a single dose of PPSV23 ≥5 years before enrollment, PCV13 elicited OPA responses that were comparable with those elicited by PPSV23 for two serotypes and higher for 10 serotypes (8).

Immunogenicity studies evaluating responses to PCV7 and PPSV23 administered in series showed a better immune response when PCV7 was administered first (9–12). An evaluation of immune response after a second pneumococcal vaccination administered 1 year after the initial study doses showed that subjects who received PPSV23 as the initial study dose had lower OPA antibody responses after subsequent administration of PCV13 than those who had received PCV13 as the initial dose followed by a dose of PPSV23, regardless of the level of the initial OPA response to PPSV23 (9). Studies evaluating the immune response after a sequence of PCV7 or PCV13 followed by PPSV23 with intervals of 2, 6, and 12 months or 3–4 years demonstrated that after the PPSV23 dose, antibody levels were higher than the pre-PCV baseline, and a noninferior response was observed when compared with post-PCV antibody levels (9–12). None of the studies were designed to evaluate the optimal interval between vaccine doses.

What is currently recommended?

In 2010, the Advisory Committee on Immunization Practices (ACIP) approved revised recommendations that all persons should be vaccinated with 23-valent pneumococcal polysaccharide vaccine (PPSV23) at age 65 years. In 2012, ACIP made recommendations for use of 13-valent pneumococcal conjugate vaccine (PCV13) and PPSV23 for adults aged ≥19 years with immunocompromising conditions.

Why are the recommendations being modified now?

PCV13 was approved by the Food and Drug Administration in late 2011 for use among adults aged ≥50 years. In June 2014, the results of a randomized placebo-controlled trial showing efficacy of PCV13 against community-acquired pneumonia among approximately 85,000 adults aged ≥65 years became available and were presented to ACIP. The evidence supporting PCV13 vaccination of adults was evaluated using the Grading of Recommendations, Assessment, Development, and Evaluation (GRADE) framework and determined to be type 2 (moderate level of evidence); the recommendation was designated as a Category A recommendation.

What are the new recommendations?

Both PCV13 and PPSV23 should be routinely administered in series to all adults aged ≥65 years. The recommendations for routine PCV13 use among adults aged ≥65 years will be reevaluated in 2018 and revised as needed. ACIP recommendations for routine use of PCV13 in adults aged ≥19 years with immunocompromising conditions, functional or anatomic asplenia, cerebrospinal fluid leak, or cochlear implants remain unchanged.

Safety of PCV13 was evaluated in approximately 6,000 PPSV23-naïve and PPSV23-experienced adults aged ≥50 years (13). Overall incidence of serious adverse events reported within 1 month of an initial study dose of PCV13 or PPSV23 did not differ between the two vaccines and ranged from 0.2% to 1.7%. From 1 to 6 months after an initial study dose, the overall incidence of serious adverse events ranged from 1.2% to 5.8% among persons vaccinated with PCV13 and 2.4% to 5.5% among persons vaccinated with PPSV23. Rates of reported serious adverse events in the treatment groups were similar among studies that enrolled PPSV23-naïve subjects and studies that enrolled PPSV23-experienced subjects. Common adverse reactions reported with PCV13 were pain, redness, and swelling at the injection site; limitation of movement of the arm in which the injection was given; fatigue; headache; chills; decreased appetite; generalized muscle pain; and joint pain. Similar reactions were observed in adults who received PPSV23 (13).

Indirect effects from PCV13 use among children, if similar to those observed after PCV7 introduction, might further reduce the remaining burden of adult pneumococcal disease caused by PCV13-types. A preliminary analysis using a probabilistic model following a single cohort of persons aged 65 years demonstrated that adding a dose of PCV13 to the current PPSV23 recommendations for adults aged ≥65 years, compared with current PPSV23 recommendations, would lead to additional health benefits (14). This strategy would prevent an estimated 230 cases of IPD and approximately 12,000 cases of community-acquired pneumonia over the lifetime of a single cohort of persons aged 65 years, assuming current indirect effects from the child immunization program and current PPSV23 vaccination coverage among adults aged ≥65 years (approximately 60%). In a setting of fully realized indirect effects assuming the same vaccination coverage, the expected benefits of PCV13 use among this cohort will likely decline to an estimated 160 cases of IPD and 4,500 cases of community-acquired pneumonia averted among persons aged ≥65 years (14).

CDC will assess the implementation and impact of the recommendation for PCV13 use among adults aged ≥65 years, including coverage with PCV13 and PPSV23, and impact of PCV13 on vaccine-type IPD burden and community-acquired pneumonia. Monitoring disease trends among adults who do not receive PCV13 might help quantify indirect effects and the long-term utility of routine PCV13 use among adults. ACIP will be updated routinely on changes in the burden of IPD and community-acquired pneumonia among adults during the next 3 years to determine the need for revisions to the adult PCV13 recommendations.

## PPSV23 in Adults

A single dose of PPSV23 is recommended for routine use in the United States among adults aged ≥65 years (15). Effectiveness of PPSV23 in preventing IPD in adults has been demonstrated, but the data on the effectiveness of this vaccine in preventing noninvasive pneumococcal pneumonia among adults aged ≥65 years have been inconsistent. PPSV23 contains 12 serotypes in common with PCV13 and 11 additional serotypes. In 2013, 38% of IPD among adults aged ≥65 years was caused by serotypes unique to PPSV23 (3). Given the high proportion of IPD caused by serotypes unique to PPSV23, broader protection is expected to be provided through use of both PCV13 and PPSV23 in series. ACIP considered multiple factors when determining the optimal interval between a dose of PCV13 and PPSV23, including immune response, safety, the risk window for protection against disease caused by serotypes unique to PPSV23, as well as timing for the next visit to the vaccination provider.

## ACIP Recommendations for PCV13 and PPSV23 Use

Both PCV13 and PPSV23 should be administered routinely in series to all adults aged ≥65 years (Box).

### Pneumococcal vaccine-naïve persons

Adults aged ≥65 years who have not previously received pneumococcal vaccine or whose previous vaccination history is unknown should receive a dose of PCV13 first, followed by a dose of PPSV23. The dose of PPSV23 should be given 6–12 months after a dose of PCV13. If PPSV23 cannot be given during this time window, the dose of PPSV23 should be given during the next visit. The two vaccines should not be coadministered, and the minimum acceptable interval between PCV13 and PPSV23 is 8 weeks.

### Previous vaccination with PPSV23

Adults aged ≥65 years who have previously received ≥1 doses of PPSV23 also should receive a dose of PCV13 if they have not yet received it. A dose of PCV13 should be given ≥1 year after receipt of the most recent PPSV23 dose. For those for whom an additional dose of PPSV23 is indicated, this subsequent PPSV23 dose should be given 6–12 months after PCV13 and ≥5 years after the most recent dose of PPSV23 (15).

### Potential Time-Limited Utility of Routine PCV13 Use Among Adults ≥65 Years

The recommendations for routine PCV13 use among adults aged ≥65 years will be reevaluated in 2018 and revised as needed.

ACIP recommendations for routine use of PCV13 in adults aged ≥19 years with immunocompromising conditions, functional or anatomic asplenia, cerebrospinal fluid leak, or cochlear implants remain unchanged (6).

## Coadministration with Other Vaccines

Concomitant administration of PCV13 and trivalent inactivated influenza vaccine (TIV) has been demonstrated to be immunogenic and safe. PCV13 can be coadministered with TIV in an adult immunization program. However, a randomized double-blind trial found slightly lower pneumococcal serotype–specific geometric mean concentrations and lower proportion achieving at least a fourfold rise in hemagglutination inhibition assay titer for one of three influenza subtypes (influenza A[H3N2]) with PCV13 plus TIV compared with PCV13 alone or TIV alone among adults aged ≥65 years (16). Currently, no data are available on coadministration with other vaccines (e.g., tetanus, diphtheria, and acellular pertussis vaccine or zoster vaccine) among adults.

## Precautions and Contraindications

Before administering PCV13, vaccination providers should consult the package insert for precautions, warnings, and contraindications. Vaccination with PCV13 is contraindicated in persons known to have a severe allergic reaction (e.g., anaphylaxis) to any component of PCV13 or PCV7 or to any diphtheria toxoid–containing vaccine.

Adverse events occurring after administration of any vaccine should be reported to the Vaccine Adverse Event Reporting System (VAERS). Reports can be submitted to VAERS online, by fax, or by mail. Additional information about VAERS is available by telephone (1-800-822-7967) or online (http://vaers.hhs.gov).

BOX **Sequential administration and recommended intervals for PCV13 and PPSV23 for adults aged ≥65 years — Advisory Committee on Immunization Practices, United States**

**Figure:**
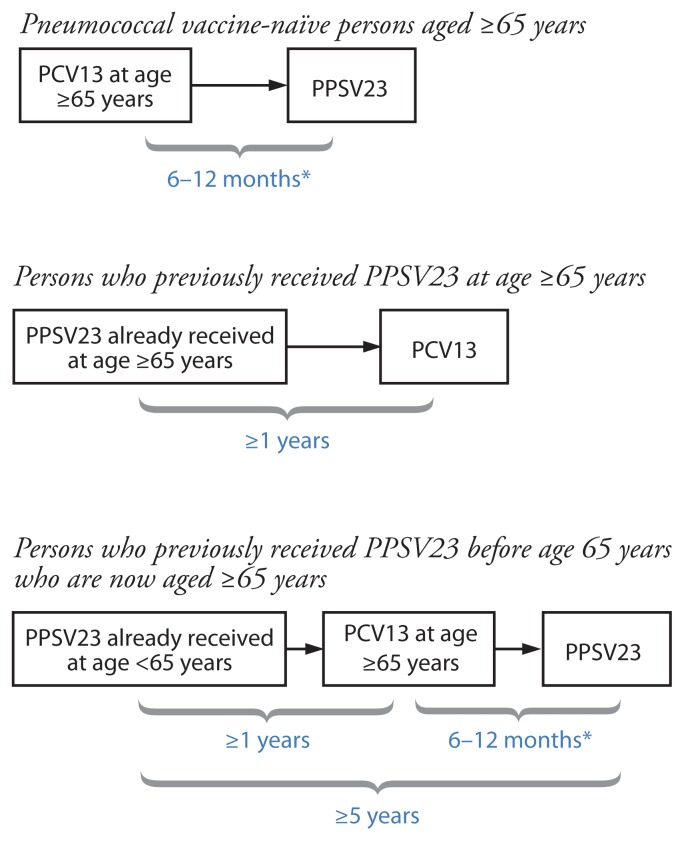

**Abbreviations:** PCV13 = 13-valent pneumococcal conjugate vaccine; PPSV23 = 23-valent pneumococcal polysaccharide vaccine.

* Minimum interval between sequential administration of PCV13 and PPSV23 is 8 weeks; PPSV23 can be given later than 6–12 months after PCV13 if this window is missed.
